# Reduced Specificity in Episodic Future Thinking in Posttraumatic Stress Disorder

**DOI:** 10.1177/2167702613495199

**Published:** 2014-03

**Authors:** Birgit Kleim, Belinda Graham, Sonia Fihosy, Richard Stott, Anke Ehlers

**Affiliations:** 1 University of Zurich, Zurich, Switzerland; 2 University College London, United Kingdom; 3 King’s College London, United Kingdom; 4 University of Oxford, United Kingdom

**Keywords:** PTSD, autobiographical memory, Autobiographical Memory Test, autobiographical memory specificity, future memory

## Abstract

Posttraumatic stress disorder (PTSD), one of the most common disorders following trauma, has been associated with a tendency to remember past personal memories in a nonspecific, overgeneral way. The present study investigated whether such a bias also applies to projections of future personal events. Trauma survivors (*N* = 50) generated brief descriptions of imagined future experiences in response to positive and negative cues in a future-based Autobiographical Memory Test. Survivors with PTSD imagined fewer specific future events in response to positive, but not to negative, cues, compared to those without PTSD. This effect was independent of comorbid major depression. Reduced memory specificity in response to positive cues was related to appraisals of foreshortened future and permanent change. Training to enhance specificity of future projections may be helpful in PTSD and protect against potentially toxic effects of autobiographical memory overgenerality.

Psychological trauma may affect autobiographical memory function in a number of ways (Brewin, Dalgleish, & Joseph, 1996; Ehlers & Clark, 2000; Moore & Zoellner, 2007). Posttraumatic stress disorder (PTSD), one of the most common disorders after trauma, has been characterized primarily as a disorder of memory (McNally, 2006). Memory difficulties in PTSD have been observed for retrieving both memories of the actual trauma (i.e., difficulties retrieving a coherent organized memory of the traumatic event) and autobiographical memories unrelated to the trauma. A bias to recall autobiographical memories in a general rather than specific way has been observed in PTSD (Kleim & Ehlers, 2008; McNally, Lasko, Macklin, & Pitman, 1995; Moore & Zoellner, 2007). This bias toward overgeneral memory (OGM) recall may serve protective functions such as attenuating painful emotions associated with past emotional memories and may develop early in life in individuals exposed to childhood trauma (Dalgleish et al., 2003; Kuyken & Brewin, 1995). Impaired executive function and ruminative thinking may further contribute to OGM (Williams et al., 2007).

This ability to retrieve specific memories from one’s past is important for visualizing one’s future, a process that has been referred to as future “mental time travel” (Tulving, 2002). The capacity to “pre-experience” future episodic events in our minds has attracted much recent scientific attention, including neuroimaging studies of the “prospective brain” (Schacter, Addis, & Buckner, 2007). These studies suggest that imagining future events recruits many of the same neural processes involved in the recall of past autobiographical memories (Szpunar, Watson, & McDermott, 2007). Imagining future episodes may depend on the ability to recall details from past events and to flexibly reconstruct these details to simulate novel future images and scenarios (Schacter et al., 2007).

This raises the question of whether an impaired ability to imagine future events may be related to posttrauma psychopathology. One of the symptoms of PTSD is a sense of foreshortened future, including a feeling that one will not live long enough to experience key events within a normal life cycle, hence affecting life in numerous areas, including the manner in which one plans for the future. The cognitive processes that underlie this problem are unclear. Negative trauma-related appraisals, such as the belief that future traumatic events are likely to happen or that one is permanently changed for the worse by the trauma, may play an important role (Ehlers & Clark, 2000). Such patterns of thinking may maintain a sense of current threat and contribute to PTSD. Given the interdependence between future projections and the recall of past autobiographical events, individuals with PTSD may be impaired in generating specific future simulations. Overgeneral, nonspecific simulation of future events has been found in depression (Williams, 1996; Williams et al., 2007), schizophrenia (D’Argembeau, Raffard, & Van der Linden, 2008), and borderline personality disorder (Kremers, Spinhoven, Van der Does, & Van Dyck, 2006) as well as in adults with traumatic grief (MacCallum & Bryant, 2011b).

The purpose of the present study is to investigate the specificity of episodic future events generated by trauma survivors. Based on previous research documenting OGM for past events in PTSD, the observation that “the extent that inability to retrieve episodes from the past hampers one’s ability to envision the future (i.e., future foreshortening)” (McNally, Litz, Prassas, Shin, & Weathers, 1994), and an initial report of future overgenerality in a small group of American combat veterans from the wars in Iraq and Afghanistan (Brown et al., 2013), we hypothesized that in a larger group of trauma survivors, PTSD would be associated with reduced specificity in imagining future events. Hampered specific future thinking may be one display of a foreshortened sense of future in those who think that the trauma has cut short their lives or that aspirations and life goals that had been important to them will no longer be reachable after the trauma. Those who believe that they have permanently changed since the trauma may find it particularly difficult to imagine specific positive personal future events. We thus hypothesized that reduced future specificity would be related to a greater perception of permanent change and foreshortened future after trauma and prior exposure to a greater number of traumas and childhood traumas. To our knowledge, this is the first study to investigate future event specificity in a mixed sample of trauma survivors and to examine some of the correlates of future event specificity.

## Method

### Participants

Assault and motor vehicle accident (MVA) survivors were recruited through flyers posted around the community and local advertisements. Inclusion criteria, assessed over the phone, included (a) experience of an assault or MVA that met the trauma A1 criterion specified in the fourth edition of the *Diagnostic and Statistical Manual of Mental Disorders* (*DSM-IV*; American Psychiatric Association [APA], 1994), (b) a minimum age of 18 years, and (c) mastery of written and spoken English to complete assessment and questionnaires. Participants with current psychosis and substance dependence as well as those who could not remember the event (e.g., because of a head injury) were excluded. Of 96 individuals interviewed on the phone, 61 met these inclusion criteria and were invited to a research session. A total of 52 participants attended, 50 of whom completed the Autobiographical Memory Test Future (AMT-f). Trauma exposure ranged between 1.5 months and 44 years prior to the study (*M* = 4.7 years). Demographic and clinical characteristics are displayed in Table 1. The PTSD and non-PTSD groups did not differ in any of these characteristics except PTSD and depression symptom severity and PTSD-characteristic appraisals of permanent change and foreshortened future.

**Table 1.** Sample Characteristics (*N* = 50)

	PTSD (*n* = 30)		Non-PTSD (*n* = 20)		
Variable	*n*	%	*n*	%	Sign. diff.
Sex (Male)	10	33.3	10	50.0	*ns*
Ethnicity (Caucasian)	14	46.7	12	60.0	*ns*
Employment status					
Employed, student	17	56.6	13	65.0	*ns*
Unemployed, retired	13	43.3	7	35.0	*ns*
Marital status					
Married/long-term relationship	15	50.0	5	25.0	*ns*
Single/divorced/other	15	50.0	15	75.0	*ns*
Trauma type					
Assault	15	50.0	9	45.0	*ns*
MVA	15	50.0	11	55.0	*ns*
Childhood abuse history					
Endorsed	14	46.7	6	33.3	*ns*
No information	0	0	2	10.0	
	*M*	*SD*	*M*	*SD*	Sign. diff.
Age (in years)	42.3	10.6	37.0	13.7	*ns*
Years of education (in years)	14.2	2.8	14.8	3.2	*ns*
Time since trauma (in years)	4.1	7.1	5.8	9.6	*ns*
Number of adult traumas	6.8	3.7	5.4	3.0	*ns*
PTSD symptom severity (PDS)	29.0	11.3	11.3	7.8	*F =* 36.2, *p* < .001
Foreshortened future (% endorsed)	11	39.4	1	5.0	χ^2^ = 7.3, *p* = .008
Permanent change (Range: 0–7)	3.6	1.9	1.8	1.3	*F* = 12.64, *p* = .001
Imagery ability (Range: 0–55)	41.41	8.40	39.26	9.13	*ns*

Note: MVA = motor vehicle accident; PDS = Posttraumatic Diagnostic Scale; PTSD = posttraumatic stress disorder.

### Measures

#### AMT-f

The AMT-f was administered individually following standard procedures. Participants saw 12 cue words (6 positive words: cheer, pleased, relieved, lively, glorious, peaceful; 6 negative words: worse, guilty, hopeless, awful, grave, ugly). Cues were derived from earlier studies (Kleim & Ehlers, 2008) and presented in random order on a computer screen. The participants’ task was to generate, and briefly describe, a specific personal event relating to the participants’ future in response to each cue word. Specific future events were explained as important or trivial events that would happen on a particular day, lasting no longer than a day. Examples of appropriate specific future events and inappropriate general events were given, and participants had the opportunity to practice the task. Participants were allowed a maximum of 1 minute to retrieve a specific future personal event for each word. If they did not provide a response in that time, this was scored as an omission and counted as a nonspecific response. Responses were tape-recorded and later transcribed and scored for the total number of first personal future events that were specific and for their trauma-relatedness. A future event was rated as trauma-related when it involved the trauma or its consequences (e.g., “When I will look into the mirror tomorrow morning and see the scar from the assault”). A psychologist with a bachelor’s degree in psychology training for a doctorate in clinical psychology rated all memories and was blind to the participants’ diagnostic status. A second independent rater scored a random sample of 35 oral AMT-f responses; there was good interrater agreement for the categorization of specific versus nonspecific responses (κ = .81).

### Structured Clinical Interviews

#### PTSD and major depression diagnoses

PTSD diagnosis was established with a standard structured clinical interview, the Clinician-Administered PTSD Scale (Blake et al., 1995). The interviewer, a trained psychologist with a bachelor’s degree in psychology, rated each of the PTSD symptoms for frequency and for intensity, each on a scale from 0 to 4. PTSD was rated as present if the participant reported the number of symptoms specified in *DSM-IV* (APA, 1994). Major depression diagnosis was assessed with the Structured Clinical Interview for *DSM-IV* (First, Spitzer, Gibbon, & Williams, 1996). Interrater reliability was high (PTSD: κ = .80; major depression: κ = .80 based on 10 interviews; 2 raters who were each uninformed as to the other rater’s diagnoses).

### Questionnaires and Other Measures

#### Permanent change

Permanent change was assessed with a four-item subscale from the Posttraumatic Cognition Inventory (PTCI; Foa, Ehlers, Clark, Tolin, & Orsillo, 1999). An example item is “I have permanently changed for the worse.” The PTCI measures trauma-related thoughts and beliefs that have been shown to discriminate well between trauma survivors with and without PTSD. It has been shown to have good internal consistency and retest reliability (α = .88 for the subscale in the current sample). The permanent change scale has been shown to correlate with OGM (Kleim & Ehlers, 2008; Schönfeld & Ehlers, 2006).

#### Trauma and childhood abuse history

Trauma history including childhood abuse was assessed with the Traumatic Life Events Questionnaire (Kubany et al., 2000), which assesses exposure to a broad range of 17 types of traumatic events. Participants were asked to indicate whether they had experienced each event. The scale has been shown to possess adequate temporal stability. Questionnaire and structured interview versions yielded similar results. For the present study, we used the sum score indexing the number of traumatic events experienced as well as endorsement of serious physical or emotional abuse as a child.

#### Verbal intelligence

The National Adult Reading Test (NART; Nelson, 1991), an established measure of verbal intelligence, requires participants to read aloud a list of 50 irregularly spelled words in order of increasing difficulty. Responses are individually scored as correct or incorrect, according to their pronunciation. The number of words read correctly composes the final score.

#### Depression symptom severity

The Beck Depression Inventory (BDI; Beck & Steer, 1987) is a widely used, standardized, and normed measure of severity of depression. The BDI asks participants to decide between four different response choices reflecting different degrees of symptom severity. Items are then scored from 0 to 3, with the sum of the item scores representing the total BDI score, ranging between 0 and 63. Internal consistency in the present study was very good (α = .93).

#### PTSD symptom severity

The Posttraumatic Diagnostic Scale (PDS; Foa et al., 1997) is a standardized and validated self-report measure of PTSD symptom severity that has been widely used with clinical and nonclinical samples of traumatized individuals. The PDS asks participants to rate 17 items regarding how much they were bothered by each of the PTSD symptoms specified in *DSM-IV* ranging from 0 (*never*) to 3 (*5 times per week or more/very severely*). Internal consistency in the present study was very good (α = .95).

### Procedure

The study was approved by the local ethics committee. Participants were recruited via flyers and screened over the phone. If they met inclusion criteria, they were invited to a research session. In the session, participants provided some details about their traumatic event, took part in a diary study regarding their intrusive traumatic memories (to be reported elsewhere), and filled in questionnaires. They then completed the AMT-f. The diagnostic interviews followed. Participants were reimbursed £50 ($97) for their time and travel expenses.

### Data Analysis

The Statistical Package for the Social Sciences (SPSS v. 15.0) was used for all analyses. Analyses of variance were calculated with the generalized linear modeling procedure, with diagnostic group as the between factor (e.g., PTSD vs. no PTSD) and AMT-f cue valence (positive vs. negative) as the within-subject factor. Spearman correlations were calculated between specificity scores, imagery ability, permanent change, foreshortened future, and verbal intelligence. The significance level was set at *p* = .05 (one-tailed for tests of directed correlational hypotheses regarding the association among specificity, appraisals of permanent change and foreshortened future, and prior trauma exposure; two-tailed for hypotheses regarding diagnostic group differences and specificity). Standard measures of effect size, that is, Cohen’s *d*, are reported for significant group differences.

## Results

### Response Latency, Omissions, and Trauma-Relatedness of AMT-f Answers

Means for response latency to first specific future personal event generated and trauma-relatedness of answers are shown in Table 2, including statistics for significant results. Participants with PTSD did not differ significantly from those without PTSD in response latency (*p* = .891). There was a significant valence effect, with faster responses to positive compared to negative cues. There was no interaction between PTSD group and valence (*p* = .583). More omissions were produced in response to negative than to positive cues, overall. For trauma-relatedness, there was a main effect of group, indicating that participants with PTSD produced more trauma-related future events than did participants without PTSD. This effect was irrespective of cue valence, as there were no main effects of valence (*p* = .101) and no interaction between diagnostic group and valence (*p* = .791).

**Table 2.** Means and Standard Deviations for AMT-f Answer Specificity, Omission, Trauma-Relatedness, and Latency as Well as Significant Main Effects and Interactions

	PTSD				Non-PTSD				
	Positive		Negative		Positive		Negative		
AMT-f aspect	*M*	*SD*	*M*	*SD*	*M*	*SD*	*M*	*SD*	Significant effects
Latency to first specific event generated	13.51	8.85	25.56	12.52	12.26	11.58	25.95	14.44	Valence main effect: N > P (*F =* 75.76, *p* < .001)
Omission	6.90	11.37	18.97	21.23	4.17	13.11	21.67	21.70	Valence main effect: P < N (*F* = 24.75, *p* < .001)
Trauma-relatedness	41.38	62.78	34.45	55.27	10.00	30.78	5.00	22.36	PTSD main effect: PTSD > non-PTSD (*F =* 4.92, *p* = .031)
Specificity of first future personal event generated	50.57	26.53	29.89	26.11	67.54	33.09	22.81	26.12	Valence main effect: P > N (*F =* 54.46, *p* < .001); Valence × PTSD: PTSD P < non-PTSD P (*F =* 7.36, *p* = .009)

Note: First specific event, omission, and trauma-relatedness in %, latency in seconds. AMT-f = Autobiographical Memory Test Future; N = response to negative cue; P = response to positive cue; PTSD = posttraumatic stress disorder.

### Future Event Specificity in Trauma Survivors With and Without PTSD

The mean specificity of future events generated by the PTSD and non-PTSD groups is shown in Table 2. The 2 (group) × 2 (cue valence) ANOVA showed no main effect of diagnostic group (*p* = .471), but a significant main effect of cue valence. Overall, participants imagined more specific future events in response to positive (almost 60% specificity) than to negative cues (28% specificity; *d* = 1.08). The interaction between cue valence and group was significant. Although the groups did not differ in generating specific future events to negative cues (*p* = .355, *d* = 0.28), participants with PTSD produced fewer specific future events in response to positive cues than did those without PTSD (*d* = 0.57). The interaction remained significant when trauma-relatedness of AMT-f answers was controlled for (*p* = .020) and when participants whose traumatic events happened more than 10 years ago were excluded (*p* = .046). Index trauma type (assault vs. MVA) and time since trauma were not significantly associated with memory specificity scores (*p* values > .278).

We also tested whether comorbid major depression was driving the low specificity to positive cues in PTSD. This was not the case. Specificity scores were almost identical between participants with PTSD and comorbid depression (*n* = 12) versus those with PTSD without comorbid depression (*n* = 17), *M* (*SD*)_PTSD + depression_ = 50.00 (27.30) versus *M* (*SD*)_PTSD without comorbid depression_ = 50.98 (27.31), *F*(1, 28) = 0.01, *p* = .924, *d* = 0.21.

### Correlates of Future Event Specificity

Greater future event specificity to positive cues in the total sample was associated with higher imagery ability (*r* = .24, *p* = .044). Specificity was negatively related to a sense of foreshortened future (*r* = −.28, *p* = .030) and to perceived permanent change since their trauma (*r* = −.25, *p* = .041). Neither of these variables was significantly associated with specificity to negative cues (all *p*s > .071). Specificity to both negative and positive cues was unrelated to verbal intelligence as assessed by the NART (*r* = .14, *p* = .173) and the number of traumatic events experienced (*p* > .455). However, childhood abuse was related to low specificity. Participants with a childhood abuse history generated fewer specific future events to positive cues than did those without a childhood abuse history (46%, *SD* = 43.83 specificity vs. 67%, *SD* = 25.97; *F* = 6.13, *p* = .017, *d* = 0.58), whereas there was no group difference for negative cues (*F* = 0.53, *p* = .470, *d* = 0.10).

## Discussion

A considerable body of literature suggests that people with PTSD tend to remember personal past events in an overgeneral rather than specific way (Moore & Zoellner, 2007). The present study investigated whether such a bias also applies to projections of future personal events. In line with previous research (Bryant, Sutherland, & Guthrie, 2007), trauma survivors with PTSD generated fewer specific future events to positive cues, but not to negative cues, than those without PTSD. A recent study by Brown et al. (2013) found reduced future specificity in American veterans in response to neutral cue words; the effect of cue valence thus needs further research and clarification. We speculate, however, that deficits in positive future episodic thinking may be particularly toxic and related to psychopathology. Blackwell and colleagues (2013) indeed found a direct association between dispositional optimism and positive future imagery and suggested positive future imagery as a cognitive target for treatment approaches to promoting optimism.

Reduced future specificity in response to positive cues was not the result of comorbid depression in our study, as participants with PTSD without depression showed the same problems in generating specific future personal events as did those with comorbid depression. Differences in specificity also remained significant after controlling for trauma-relatedness of the memories, thus ruling out the explanation that the results are from preferential access to trauma memories as compared to other memories in PTSD. The relationship between low future specificity and posttrauma psychopathology was further supported by the result that PTSD-related cognitions, a sense of foreshortened future, and perceived permanent change since the trauma were associated with low specificity in response to positive cues, although these findings are preliminary, awaiting replication in future studies.

Participants in this study were faster to respond and produced fewer omissions to positive cues than to negative cues. These findings are consistent with the notion of a pervasive positive bias in expectations of the future (Carver, Scheier, & Segerstrom, 2010). Imagining the future in positive ways can be adaptive, and people generally seem to be better able to imagine their future in positive ways than in negative ways (Hoorens, Smits, & Shepperd, 2008). This may not be the case for people with PTSD, who had specific difficulties generating future personal events in response to positive cues compared to those without PTSD; specificity was reduced in PTSD by almost 20%. This is in accord with the finding that people with PTSD may view their anticipated future self less favorably than their pretrauma self (Brown, Buckner, & Hirst, 2011). Specificity to negative cues, on the other hand, did not differ significantly between survivors with versus without PTSD (see also Kremers et al., 2006). OGM for past autobiographical memories in PTSD, however, appears to be largely independent of cue valence in PTSD (Moore & Zoellner, 2007).

Impaired executive control has been suggested as a possible explanation for difficulties in accessing specific autobiographical material (Dalgleish et al., 2007). As future simulation relies on past memory retrieval and its flexible recombination, it is taxing for memory systems, and poor executive control in PTSD may thus lead to low specificity. However, impaired executive control alone cannot explain the difficulties with future personal event generation in PTSD, as we found a specific effect in response to positive cues, whereas executive control deficits should affect answers to both cue types. Moreover, we did not find any significant associations between our measure of verbal intelligence (NART) and memory specificity.

Another explanation for the reduced future specificity in PTSD in response to positive cues is that trauma survivors with PTSD may become “stuck” in their prior traumatic experience (Holman & Silver, 1998) and have difficulty maintaining a future orientation in the aftermath of their trauma. Such temporal orientation is pivotal in providing orientation and structuring people’s view of themselves in the world (Holman & Silver, 1998). Our results are in accord with these prior findings. Individuals without PTSD were better able to project themselves into the future in response to positive cues. These individuals may be better able to shift attention away from distressing past life events and instead focus on concrete positive future possibilities. This seems also in line with our finding that those who felt that they had permanently changed for the worse since the trauma and those who felt that their future had been cut short were less specific to positive cues. This parallels findings that these changes in self-perception also correlate with past OGM (Schönfeld & Ehlers, 2006). They may contribute to the effect of “being stuck in the past” and to the difficulty in projecting oneself into the future in a positive way. In addition to a lack of future projections, people with PTSD may be characterized by impoverished and trauma-oriented future imaginings, hence leading to a lack of future specificity to positive events.

We also assessed the relation of prior trauma exposure and specificity of future projections. The number of past traumatic events was unrelated to future specificity, suggesting that one trauma may be sufficient in producing an overgeneral bias. However, because most participants reported several traumatic events, the lack of a correlation may also be the result of a ceiling effect. Childhood abuse was, however, related to future memory specificity. Although we did not assess whether childhood abuse was part of the participants’ current re-experiencing symptoms, we found that those with childhood trauma exposure were less specific in response to positive cues than were those without. Childhood trauma has been emphasized as a possible pathway to the development of overgeneral autobiographical memory bias (Brennen et al., 2010; Williams et al., 2007). It is possible that an overgeneral bias for projections of the personal future may have already developed early in development in these individuals.

Our results have clinical implications. Training in concrete and specific past autobiographical remembering has been shown to decrease depressive symptoms in dysphoric individuals (Watkins, Baeyens, & Read, 2009). An intriguing question would be whether successful training of past memory specificity also leads to increased future specificity, or whether alternative ways of training are warranted. Recent cognitive therapeutic innovations include, for instance, a training program in imagining positive events, originally developed as a “cognitive vaccine” against depressed mood (Holmes, Lang, & Sha, 2009). Past memory specificity training has so far been reported mostly for individuals with depression (e.g., Neshat-Doost et al., 2013; Serrano et al., 2004), and future research needs to test whether these results extend to future event specificity and to individuals with PTSD. Moreover, it would be of interest whether future episodic thinking improves during treatment for PTSD. Trauma-focused cognitive-behavioral therapy (CBT) for PTSD, for instance, may help identify and modify thoughts that render some trauma survivors prone to feel their future has been curtailed and that they have permanently changed following the trauma (Ehlers, Clark, Hackmann, McManus, & Fennell, 2005). Interestingly, a recent study found that past OGM reduced during CBT in individuals with complicated grief and that grief symptom reductions were associated with increased specific retrieval to positive cues following treatment (MacCallum & Bryant, 2011a).

The current study is not without limitations. First, our data are cross-sectional, and it remains unclear whether the deficit in future specificity in response to positive cues is a consequence of PTSD or a factor involved in the development and maintenance of the disorder (Bryant et al., 2007). Second, our results do not elucidate the mechanisms of the association between future specificity and PTSD. A next step would be to test candidate mediators of this effect in a longitudinal design. Third, further phenomenological characteristics of the future events produced by our participants may be of importance. Some autobiographical memory characteristics that have shown associations with psychopathology in previous research, such as the emotional valence of the generated events, vantage perspective, time frame, or the content of the imagined events, may also influence the association between PTSD and specificity and should be assessed by future studies. Fourth, as applicable to the standard AMT (Griffith et al., 2012), the AMT-f must be examined in further samples with respect to its psychometric properties, including reliability and validity, taking into account potential variability in methodology across the studies that use it. Such examinations are of particular importance before the AMT-f is administered and used in clinical contexts. Finally, because of the relatively small sample size, results on the correlates of future event specificity were based on correlations in the overall sample and should thus be replicated in future studies.

To the best of our knowledge, the present study is among the first investigations into future mental time travel in trauma survivors with and without PTSD. Future studies are needed to replicate the effect, including prospective studies investigating future specificity pretrauma and shortly after trauma, and its relationship to posttrauma adaptation. Further studies into the association between past and future memory overgenerality are also needed. Future research could seek to assess additional factors, including potentially protective factors that promote future simulation capacity. The results could usefully inform on what may protect against the potentially toxic effects of overgenerality in past autobiographical memory and in future episodic simulation.
